# Do polluting enterprises contribute to cancer?—Empirical evidence from tumor registry data in China

**DOI:** 10.3389/fpubh.2025.1615439

**Published:** 2025-08-25

**Authors:** Jiwen Liu, Bingquan Lin

**Affiliations:** ^1^Department of Economics and Management, Jiangsu College of Administration, Nanjing, China; ^2^School of Geographic Sciences, East China Normal University, Shanghai, China

**Keywords:** polluting enterprises, cancer incidence rate, public health, PM2.5, government regulation polluting enterprises, government regulation

## Abstract

This study utilizes data from the China Cancer Registry Annual Report and the “Qichacha” database to construct a time-region regression model based on panel data, aiming to explore the impact of polluting enterprises on regional cancer incidence rates between 2000 and 2008. The empirical analysis reveals that polluting enterprises significantly increase cancer incidence rates among both male and female populations, with a particularly marked effect on lung cancer. Heterogeneity analysis shows that the effect is most pronounced in the eastern and coastal regions, followed by the central and northeastern areas, with minimal impact in the western and inland regions. There may be a threshold effect in the influence of polluting enterprises on cancer incidence. Additionally, local government environmental regulations can mitigate the negative health impacts of industrial pollution. Mechanism analysis indicates that polluting enterprises influence regional public health through environmental pollution, while also highlighting potential disparities in urban-rural medical compensation. The findings provide empirical support and mechanism validation for understanding the impact of polluting enterprises on public health, offering valuable insights for the “Healthy China” strategy and environmental governance policies.

## 1 Introduction

Since the Industrial Revolution, urbanization, industrialization, and economic development have significantly increased global energy consumption and waste generation. In recent years, the trade-off between economic growth and environmental sustainability has become particularly critical in rapidly urbanizing developing countries such as China and India, where both industrial productivity and pollution levels are on the rise (1, 2).

As the world's largest developing country and one of the largest emitters of pollutants, China plays a pivotal role in global environmental governance. The rapid economic development and industrialization may significantly increase the cancer burden in China. According to the 2024 National Cancer Report published by the National Cancer Center, in 2022, there were 19.96 million new cancer cases globally. Of these, ~4.8 million new cancer cases were reported in China, accounting for 24% of the global total. Cancer remains the leading public health challenge in China.

From 2000 to 2008, China's economy transitioned from joining globalization to rapid development, during which many regions relied on polluting industries for swift economic growth. In the 2008 Environmental Performance Index report, China ranked 121st out of 163 evaluated countries, which reflects the environmental pollution issues arising from China's rapid industrialization. As polluting industries expanded across domestic regions, the resulting conflicts between economic growth and public health became more pronounced. Between 2004 and 2013, reports of “cancer villages” became increasingly frequent. For instance, in 2005, Dongxing Village in Yancheng, Jiangsu Province, was reported to have a chemical plant named Julong Chemical, which discharged large amounts of toxic gases and wastewater into the air and water sources. As a result, over 100 residents of the village developed cancer between 2000 and 2005. By the mid-2010s, at least 459 cancer villages had been confirmed. Beyond the directly reported “cancer village” phenomenon, the question remains: Are there lingering “aftereffects” caused by polluting enterprises for local residents? This is the issue that this study aims to explore.

Polluting enterprises, as key contributors to environmental pollution, generate PM2.5 through the emission of exhaust gases and further exacerbate PM2.5 concentrations through the discharge of industrial wastewater (3, 4). The impact of environmental pollution on public health has become increasingly pronounced (5, 6). A substantial body of authoritative medical literature has confirmed that long-term exposure to PM2.5 significantly increases the risk of developing various cancers, including lung cancer, breast cancer, and colorectal cancer (7–9). Most case studies to date have found a significant impact of polluting enterprises on cancer incidence among local residents (10–13). However, there is a lack of large-scale empirical studies on this issue. Therefore, this paper empirically investigates the impact of polluting enterprises on the health burden of residents, particularly cancer incidence, using economic methods.

As China experienced rapid economic growth from 2000 to 2008, polluting enterprises played a significant role in regional prosperity. However, this growth may have come at the expense of residents' health, particularly regarding cancer rates. This study aims to explore the impact of polluting enterprises on local health, using cancer incidence data from 1,797 regions between 2008 and 2016, sourced from the China Cancer Registry Annual Report. Data on polluting enterprises were gathered from the “Qichacha” database, and pollution density was calculated to analyze its relationship with cancer rates.

The empirical analysis reveals that polluting enterprises significantly increase cancer incidence rates among both male and female populations, particularly with a marked effect on lung cancer, thereby exacerbating the public health burden. This effect is more pronounced in eastern and coastal regions, followed by central and northeastern areas, with negligible impacts in western and inland regions. Additionally, there may be a threshold effect in the influence of polluting enterprises on cancer incidence. Local government environmental regulations can mitigate the negative health impacts of industrial pollution and reduce the cancer incidence risk associated with polluting enterprises. The potential impact on male cancer incidence is greater than that on females, likely related to gender differences in occupational choices. This study empirically demonstrates that polluting enterprises influence regional public health through environmental pollution, while also identifying potential disparities in medical compensation. The findings provide empirical support and mechanism validation for understanding the impact of polluting enterprises on public health.

This paper makes the following contributions: first, Previous studies often examine the relationship between regional industrialization and environmental pollution, or the impact of environmental pollution on residents' health, in isolation. Few have integrated these two aspects to investigate how regional industrialization affects public health outcomes. This study bridges these research domains by introducing environmental pollution as a mediating mechanism, offering a novel perspective on the link between industrialization and health. Second, This study empirically demonstrates how polluting enterprises contribute to environmental pollution, thereby revealing the threshold effects and underlying mechanisms through which such enterprises influence regional cancer incidence. This approach goes beyond the case-based analyses commonly found in existing literature. Third, Taking into account geographic and environmental factors, this study adopts a two-way fixed effects regression model to analyze this issue within the framework of health economics. By evaluating the impact of polluting enterprise density on regional cancer incidence, the study significantly improves the accuracy and robustness of its findings. Finally, This study systematically investigates the effects and mechanisms of polluting enterprises on cancer incidence. The results offer meaningful implications for China's future policy decisions regarding investment attraction and industrial planning, and provide valuable references for other emerging economies pursuing industrialization-led growth.

This paper is organized as follows:

Chapter 1 provides the Introduction, outlining the research background and significance.

Chapter 2 presents the Hypothesis Development, where we construct the underlying causal framework for our analysis.

Chapter 3 covers Materials and Methods, detailing the measurement of key variables, data sources, and the construction of the regression equations.

Chapter 4 offers the Empirical Analysis, including baseline regression, robustness checks, heterogeneity analysis, endogeneity analysis, and mechanism analysis. This section also further explores the differences in medical compensation.

Chapter 5 is the Discussion, in which we summarize the key findings, provide relevant policy recommendations, and discuss the study's limitations as well as directions for future research.

## 2 Hypothesis development

Existing research has emphasized the strong correlation between socioeconomic development and disease incidence. As integral components of the regional economy, polluting enterprises can substantially influence cancer incidence rates by causing significant environmental degradation. Specifically, during their production processes, these enterprises emit large volumes of hazardous waste gases, wastewater, and other pollutants, leading to persistent environmental contamination. Prolonged exposure to such polluted environments increases residents' risk of developing cancer, establishing a clear causal pathway from industrial pollution to adverse health outcomes. In contrast, non-polluting enterprises produce minimal environmental harm, and as such, their impact on regional cancer incidence is negligible. Based on the above theoretical analysis, this study proposes the following hypotheses.

Hypothesis 1: Polluting enterprises have a more significant impact on regional cancer incidence rates compared to non-polluting enterprises.

The presence of polluting enterprises significantly increases the level of environmental pollution, particularly fine particulate matter (PM2.5), which is considered one of the most important pollutants in environmental pollution. A large body of research has shown that long-term exposure to high concentrations of PM2.5 can exacerbate the burden of diseases, including cancer (8, 9). Therefore, this study uses PM2.5 concentration as a measure of environmental pollution and hypothesizes that polluting enterprises, by exacerbating environmental pollution and increasing regional PM2.5 concentrations, significantly influence cancer incidence rates. Based on the above theoretical analysis, this study proposes the following hypotheses:

Hypothesis 2: Polluting enterprises exacerbate environmental pollution, increasing regional PM2.5 concentrations, which in turn raises cancer incidence rates in the region.

There is a nonlinear relationship between the reduction in air pollution and improvements in children's health (10), suggesting that once pollution levels reach a certain threshold, its impact on health becomes more significant. Based on this perspective, the density of polluting enterprises might also exhibit a threshold effect on cancer incidence rates. Given the dual role of polluting enterprises in local economic growth and environmental pollution, the study of this threshold effect is of great value. Specifically, when pollution reaches a critical threshold, cancer incidence may significantly increase, while areas with lower pollution density might experience less impact. Based on the above theoretical analysis, this study proposes the following hypotheses:

Hypothesis 3: there may be a threshold effect in the relationship between polluting enterprises and cancer incidence, such that once pollution reaches a certain level, it has a significant positive impact on cancer incidence.

At the same time, government environmental regulations play a crucial role in pollution control and protecting residents' health. In China, a country with strong governmental capacity, policy enforcement is especially important. Previous studies have shown that measures such as China's air control (11), national health city campaign (12), and Green credit policy (13) can help improve residents' health. Therefore, government environmental regulations may significantly mitigate the negative health impacts of polluting enterprises on cancer incidence. Based on the above theoretical analysis, this study proposes the following hypotheses:

Hypothesis 4: government environmental regulations play an important role in mitigating the impact of polluting enterprises on cancer incidence. Regions with higher levels of regulation may be more effective in suppressing the health impacts of polluting enterprises on cancer rates.

## 3 Materials and methods

### 3.1 Dataset

#### 3.1.1 Dependent variable

The China Cancer Registry Annual Report, published by the National Cancer Center, is an official statistical yearbook. Its data comes from tumor registries across the country, covering a broad range of areas and ensuring high data quality. It is widely used in cancer epidemiology research and the formulation of public health policies. Since the data published in the China Cancer Registry Annual Report typically corresponds to cancer incidence data from registry points 3 years earlier, and cancer data from specific registry points has not been made public since 2020, this study uses data from the China Cancer Registry Annual Report for the years 2011–2019 to obtain cancer incidence rates from 2008–2016. The cancer incidence rate is measured in cases per 100,000 people. Between 2008 and 2016, there were originally 2,089 registry points. This study used Python's Chinese text segmentation module to process the annual report texts, screening out regions like Tibet, Xinjiang Production and Construction Corps, and areas with significant data gaps. In the end, data from 1,797 registry points during 2008–2016 were obtained and used as the dependent variable for this study.

#### 3.1.2 Core explanatory variable

The identification of polluting enterprises typically involves first defining pollution-intensive industries and then considering the enterprises within these industries as polluting enterprises. Pollution-intensive industries refer to industries that, if not effectively managed during production, directly or indirectly generate large amounts of pollutants, which can harm humans, animals, and plants, thereby contributing to environmental degradation. To identify polluting enterprises, the common approach is to first define pollution-intensive industries and then classify all enterprises within these industries as polluting enterprises (14, 15).

The identification of polluting enterprises typically involves first defining pollution-intensive industries and then considering the enterprises within these industries as polluting enterprises. Pollution-intensive industries refer to industries that, if not effectively managed during production, directly or indirectly generate large amounts of pollutants, which can harm humans, animals, and plants, thus contributing to environmental degradation. To identify polluting enterprises, common methods include first defining pollution-intensive industries and then classifying all enterprises within these industries as polluting enterprises. There are three main methods for defining polluting enterprises in academic literature:

Legal Method: this relies on official documents issued by the government and relevant departments to identify polluting industries. Examples of such documents include the Ministry of Environmental Protection's Notice on Environmental Protection Inspections for Listed Companies Applying for IPOs or Refunding and the Environmental Information Disclosure Guidelines for Listed Companies, as well as the First National Pollution Source Census Plan published by the State Council (16).

Total or Intensity Method: this method sets pollution emission thresholds and selects polluting industries based on the total amount of pollution or the intensity of pollution (17).

Cost Method: this method selects polluting industries based on the proportion of pollution control costs within the total costs (18).

This study integrates the above methods and uses official documents issued by the government and relevant ministries. For industry classification, it adopts the 2017 National Economic Industry Classification (GB/T 4754-2017) standard. The data on polluting enterprises primarily comes from the information collected between 2001 and 2018 on the “Qichacha” enterprise credit information inquiry platform (www.qcc.com, henceforth referred to as the “Qichacha Database”).

The identification of polluting enterprises is first carried out by classifying the enterprises based on their industry affiliation, and then determining whether they are polluting enterprises. Specifically, the industry codes and names for the polluting enterprises identified in this study are as follows: (6) Coal mining and washing industry, (7) Petroleum and natural gas extraction industry, (8) Ferrous metal mining and selection industry, (9) Non-ferrous metal mining and selection industry, (10) Non-metallic mineral mining and selection industry, (11) Mining support activities, (12) Other mining industries, (13) Processing of agricultural and sideline products, (14) Food manufacturing industry, (15) Beverage, alcoholic drinks, and refined tea manufacturing industry, (16) Tobacco products industry, (17) Textile industry, (18) Textile, apparel, and accessories industry, (19) Leather, fur, feathers and their products, and footwear manufacturing industry, (20) Wood processing and wood, bamboo, rattan, palm, and grass products industry, (21) Furniture manufacturing industry, (22) Paper and paper products manufacturing industry, (23) Printing and reproduction of recorded media industry, (24) Manufacturing of cultural, educational, industrial, and sports equipment, (25) Petroleum, coal, and other fuel processing industry, (26) Manufacture of chemical raw materials and chemical products, (27) Pharmaceutical manufacturing industry, (28) Manufacture of chemical fibers, (29) Rubber and plastic products industry, (30) Manufacture of non-metallic mineral products, (31) Ferrous metal smelting and rolling processing industry, (32) Non-ferrous metal smelting and rolling processing industry, (33) Metal products industry, (34) General equipment manufacturing industry, (35) Special equipment manufacturing industry, (36) Automobile manufacturing industry, (37) Manufacturing of railway, shipbuilding, aerospace, and other transportation equipment, (38) Manufacturing of electrical machinery and equipment, (39) Manufacturing of computers, communications, and other electronic equipment, (40) Manufacturing of instruments and meters, (41) Other manufacturing industries, (42) Comprehensive utilization of waste resources industry, (43) Repair of metal products, machinery, and equipment, (44) Electricity and heat production and supply industry, (45) Gas production and supply industry, (46) Water production and supply industry.

Building on this, the study considers the latency period of cancer incidence (19) and the delayed effects of exposure to polluted environments, such as high concentrations of PM2.5 (20). Prior studies have used a 5-year lag to examine the relationship between environmental pollution and cancer incidence, and have found that such associations persist even with a 10-year latency period (21). Building on this evidence, and accounting for the delay between the onset of production by polluting enterprises and their environmental impact, this study adopts an average lag period of 8 years as the baseline for the regression analysis. Accordingly, to assess the impact of polluting enterprises on the environment and the subsequent delayed effects on cancer incidence, the density of polluting enterprises 8 years prior to the cancer registry year is used as the key explanatory variable. The pollution enterprises are then matched to the corresponding city or district through regional selection, allowing for the determination of the number of polluting enterprises at each registry point. Furthermore, the China City Statistical Yearbook and China County Statistical Yearbook provide data on the built-up area for each registry point, and for regions with missing data, satellite positioning technology is used to estimate the built-up area. Finally, the pollution enterprise density is calculated by dividing the number of polluting enterprises at the registry point by the built-up area, and this is used as the core explanatory variable in this study.

In addition, the method for calculating enterprise size density (ESD) is as follows: enterprises are classified into large (66%−100%), medium (33%−66%), and small (0%−33%) categories based on their registered capital, and assigned rankings of 1, 2, and 3, respectively. By weighting and summing the number of enterprises in each size category, the result is then divided by the built-up area to obtain the enterprise size density. Furthermore, this study extracts manufacturing enterprises from the polluting enterprises based on industry codes for further analysis. In addition, the study classifies heavy and light polluting enterprises based on the total volume or intensity of pollution in different industries (17). It then separately calculates the density of manufacturing enterprises, heavy polluting enterprises, and light polluting enterprises. Separately calculates the manufacturing industry density, heavy pollution enterprise density, and light pollution enterprise density.

**Figure 1:**
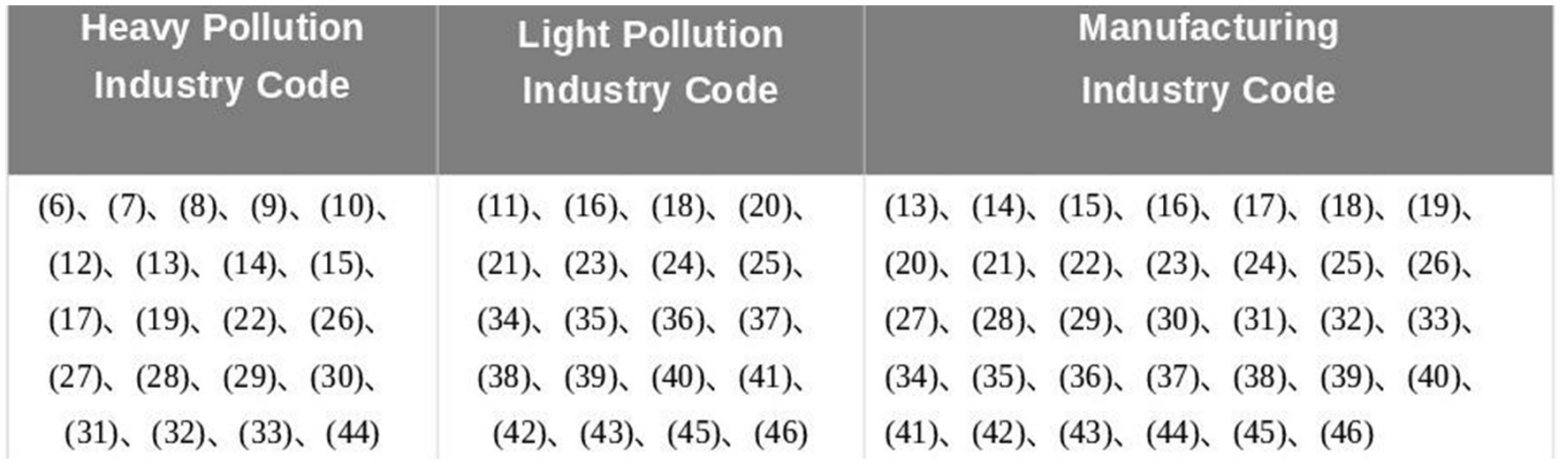
Detailed classification table of polluting enterprises by industry codes.

The above detailed industry classification standards is shown in Figure 1.

#### 3.1.3 Control variables

To exclude potential confounding effects of the impact of polluting enterprises on economic development, which could, in turn, influence cancer incidence rates, this study considers environmental factors in the selection of control variables. In a green environment, vegetation can reduce the spread of air pollutants by trapping small particles (22). Furthermore, existing studies have found that exposure to green spaces may have a potentially beneficial association with the incidence of various cancers (23). Therefore, this study specifically focuses on and controls for environmental factors in the region where each registry point is located. Specifically, this study controls for multiple environmental variables related to land use types, including the coverage area of agricultural land, forests, green spaces, water bodies, and undeveloped land. To ensure comparability across different regions, the coverage area of each land use type is divided by the built-up area of the registry point, calculating the coverage level of each land type per unit of built-up area. This approach effectively eliminates potential biases arising from differences in built-up area size. All relevant land cover data are sourced from the 30 m Annual Land Cover Grid Data Set for China, published by Wuhan University. This high-resolution dataset provides annual coverage information for various land types across the country, offering reliable spatial data support for the analysis.

Table 1 presents the descriptive statistics for the main variables.

**Table 1:** Descriptive statistics of basic variables.

**Variable name**	**Variable meaning**	***N***	**Mean**	**Min**	**Max**
MCIR	Overall cancer incidence rate for males (per 100,000 people)	1,797	296.99	127.42	811.97
FCIR	Overall cancer incidence rate for females (per 100,000 people)	1,797	228.25	48.17	532.76
PED	Number of polluting enterprises per built-up area (enterprises/km^2^)	1,797	0.67	0.01	7.07
NPED	Number of non-polluting enterprises per built-up area (enterprises/km^2^)	1,797	490.56	1.60	12,898.92
HPED	Number of heavy polluting enterprises per built-up area (enterprises/km^2^)	1,797	0.52	0.00	4.51
LPED	Number of light polluting enterprises per built-up area (enterprises/km^2^)	1,797	0.16	0.00	2.90
MED	Number of manufacturing enterprises per built-up area (enterprises/km^2^)	1,797	0.62	0.00	6.93
Atmospheric PM2.5	Annual average PM2.5 concentration (μg/m3)	1,796	52.59	5.55	112.92
Surface PM2.5	Annual average surface PM2.5 concentration (μg/m3)	1,762	5.87	1.09	43.65
Medical Level	Number of hospital beds (number of beds)	1,797	14,805.79	70.00	81,937.00
Per-Cropland	Agricultural land coverage area per built-up area (%)	1,797	35.56	0.17	293.37
Per-Forest	Forest coverage area per built-up area (%)	1,797	44.87	0.00	1,280.74
Per-Grassland	Green space coverage area per built-up area (%)	1,797	16.57	0.00	1,029.98
Per-Water	Water body coverage area per built-up area (%)	1,797	1.77	0.00	27.50
Per-Impervious	Undeveloped land coverage area per built-up area (%)	1,797	4.49	0.00	45.58

### 3.2 Method

To examine the impact of polluting enterprises on cancer incidence rates, this study constructs the following econometric model as the baseline model for empirical testing:


(1) $$\begin{array}{l} {\text{Y}}_{\text{i},\text{t}}={\text{β}}_{0}+{\text{β}}_{1}\cdot \text{PollutionDensit}{\text{y}}_{\text{i},\text{t}-8}+\text{γ}{\text{X}}_{\text{i},\text{t}}+{\text{μ}}_{\text{i}}+{\text{λ}}_{\text{t}}+{\text{ϵ}}_{\text{i},\text{t}} \end{array}$$

Where *i* and *t* represent the registry point region and year, respectively; *Y*_*i, t*_ is the cancer incidence rate at registry point *i* in year *t*, *Density*_*i, t*−8_ is the pollution enterprise density 8 years prior at registry point *i*, *X*_*i, t*_ represents other control variables, μ_*i*_ is the region fixed effect, λ_*t*_ is the year fixed effect, and ϵ_*i, t*_ is the random error term. The same as below

To examine the impact of polluting enterprises on environmental pollution, this study constructs the following econometric model as the mechanism analysis model for empirical testing:


(2) $$\begin{array}{l} \text{PM}2.5_{\text{i},\text{t}}={\text{α}}_{0}+{\text{α}}_{1}\cdot \text{PollutionDensit}{\text{y}}_{\text{i},\text{t}}+\text{γ}{\text{X}}_{\text{i},\text{t}}+{\text{μ}}_{\text{i}}+{\text{λ}}_{\text{t}}+{\text{ϵ}}_{\text{i},\text{t}} \end{array}$$

Where *i* and *t* represent the registry point region and year, respectively; *PM*2.5_*i, t*_ is the annual average surface PM2.5 concentration at registry point *i* in year *t*, measured in micrograms per cubic meter; The annual average surface PM2.5 concentration at the registry points is derived from satellite inversion data processed by NASA.

To examine the potential impact of medical compensation from polluting enterprises, this study constructs the following econometric model for empirical testing:


(3) $$\begin{array}{l} \text{MedicalLeve}{\text{l}}_{\text{i},\text{t}}={\text{γ}}_{0}+{\text{γ}}_{1}\cdot \text{PollutionDensit}{\text{y}}_{\text{i},\text{t}}+\text{γ}{\text{X}}_{\text{i},\text{t}}+{\text{μ}}_{\text{i}}+{\text{λ}}_{\text{t}}\\ \;+{\text{ϵ}}_{\text{i},\text{t}} \end{array}$$

*MedicalLevel*_*i, t*_ is represented by the number of hospital beds available in the region; The number of hospital beds is obtained from the China City Statistical Yearbook and the China Regional Statistical Yearbook.

## 4 Empirical results

### 4.1 Baseline regression

This study first constructs a time-region fixed panel model based on the overall cancer incidence rate at the registry points and performs regression estimation with robust standard errors. The regression results are shown in Table 2. Columns (1)–(2) and columns (3)–(4) correspond to the cases where the dependent variable is the overall male cancer incidence rate and the overall female cancer incidence rate, respectively. It can be seen that there is a significant positive correlation between regional pollution enterprise density and both male and female overall cancer incidence rates. Specifically, when controlling for regional environmental conditions, for every increase of 1 enterprise per km^2^ in regional pollution enterprise density, the male overall cancer incidence rate and female overall cancer incidence rate at the registry point will increase by 13.867 (per 100,000 people) and 8.565 (per 100,000 people), respectively. The positive correlation for male cancer incidence is statistically significant at the 1% level, while the positive correlation for female cancer incidence is statistically significant at the 5% level. Overall, the regression results suggest that polluting enterprises have a significant positive impact on regional cancer incidence rates.

**Table 2:** Baseline regression.

**Variable**	**(1)**	**(2)**	**(3)**	**(4)**
	**MCIR**	**MCIR**	**FCIR**	**FCIR**
PED	12.077^**^	13.867^***^	8.076^**^	8.565^**^
	(2.200)	(2.591)	(1.992)	(2.156)
NPED	−0.003	0.000	0.002	0.003
	(−1.143)	(0.225)	(1.158)	(1.129)
Time fixed effects	Y	Y	Y	Y
Region fixed effects	Y	Y	Y	Y
Control variables included	N	Y	N	Y
*N*	1,797	1,797	1,797	1,797

^*^, ^**^, ^***^ represent statistical significance levels of 10%, 5%, and 1%, respectively. The values in parentheses are t-values (the same applies to the following tables unless otherwise specified).

To further verify that it is polluting enterprises, rather than all enterprises, that contribute to the increase in regional cancer incidence rates, this study utilizes enterprise data from the Qichacha database. Enterprises are classified by industry and matched with non-polluting enterprises at the registry points. Under the same control conditions (controlling for regional environmental conditions and region-year fixed effects), a regression analysis is conducted on the overall regional cancer incidence rate, and the results are shown in Table 2. The results indicate that non-polluting enterprises do not have a significant impact on regional cancer incidence rates, further confirming the key role played by polluting enterprises in this process.

Baseline results are shown in Table 2.

### 4.2 Robustness checks

#### 4.2.1 Modification of lagged years

To ensure the robustness of our baseline regression results, this study employs a dynamic lag analysis approach to examine multiple lag periods for the core explanatory variable—polluting enterprise density (PED). Specifically, building upon the 8-year lag period used in the baseline regression, we further investigate lag periods of 6, 7, 9, and 10 years. The regression results demonstrate that: under both the 7-year and 9-year lag specifications, polluting enterprise density shows statistically significant effects (*p* < 0.1) on cancer incidence rates regardless of whether control variables are included, with coefficient directions consistent with the baseline regression; In contrast, the 6-year and 10-year lag models fail to show statistically significant effects (*p* > 0.1) of polluting enterprise density.

These findings confirm the appropriateness of selecting an 8-year lag period for our baseline regression. From the perspective of polluting enterprises' production cycles, the complete process—from factory construction and operation to pollutant accumulation and ultimately observable carcinogenic effects on human health—typically requires ~8 years of cumulative exposure. This lagged effect aligns with the biological mechanisms of environmentally-induced diseases and is supported by existing literature on the relationship between pollution exposure and cancer latency periods (24).

#### 4.2.2 Replacing the core explanatory variable

To ensure the robustness of the baseline regression results, this study conducts further regression analyses by replacing the core explanatory variable with enterprise size density and manufacturing enterprise density, as well as by excluding municipalities from the sample. The regression results show that when using pollution enterprise density as the core explanatory variable, its impact on the overall male cancer incidence rate remains significant at the 5% level, while its impact on the overall female cancer incidence rate is significant at the 10% level. Furthermore, when manufacturing enterprise density is used as the core explanatory variable, the impact on both male and female overall cancer incidence rates remains significant at the 5% level.

#### 4.2.3 Excluding municipality samples

After excluding municipalities, the regression results show that the impact of pollution enterprise density on both male and female overall cancer incidence rates remains significant at the 5% level. These findings suggest that the positive impact of pollution enterprise density on regional overall cancer incidence rates is highly significant and remains stable across different model specifications.

The above robustness regression results are shown in Table 3.

**Table 3:** Estimation results of robustness checks (1).

**Variable**	**(1)**	**(2)**	**(3)**	**(4)**
	**MCIR**	**MCIR**	**FCIR**	**FCIR**
PED_6	6.946	8.215	3.721	4.116
	(1.374)	(1.623)	(0.821)	(0.901)
PED_7	10.984^**^	12.519^***^	7.282^*^	7.720^*^
	(2.207)	(2.643)	(1.803)	(1.948)
PED_9	12.534^**^	14.334^***^	8.712^**^	9.261^**^
	(2.217)	(2.587)	(1.973)	(2.120)
PED_10	6.878	8.147	3.964	4.374
	(1.213)	(1.384)	(0.802)	(0.869)
Time fixed effects	Y	Y	Y	Y
Region fixed effects	Y	Y	Y	Y
Control variables included	N	Y	N	Y
*N*	1,797	1,797	1,797	1,797
ESD	6.615^**^	7.655^**^	4.673^*^	4.918^*^
	(2.043)	(2.402)	(1.821)	(1.927)
MED	14.379^**^	15.933^***^	9.393^**^	9.801^**^
	(2.422)	(2.739)	(2.156)	(2.288)
Time fixed effects	Y	Y	Y	Y
Region fixed effects	Y	Y	Y	Y
Control variables included	N	Y	N	Y
*N*	1,797	1,797	1,797	1,797
PED	12.208^**^	13.965^**^	9.642^**^	10.015^**^
(Excluding municipalities)	(2.205)	(2.575)	(2.321)	(2.452)
Time fixed effects	Y	Y	Y	Y
Region fixed effects	Y	Y	Y	Y
Control variables included	N	Y	N	Y
*N*	1,734	1,734	1,734	1,734

^***^p < 0.01, ^**^p < 0.05, ^*^p < 0.1.

#### 4.2.4 Changing the econometric model

In the baseline regression, a two-way fixed effects OLS model was used to regress cancer incidence rates. This section verifies the robustness of the regression results by changing the econometric model. First, male and female cancer incidence rates are divided by 100,000 to obtain male cancer probabilities (MCP) and female cancer probabilities (FCP). A Generalized Estimating Equation (GEE) is then used to estimate the impact of pollution enterprise density, manufacturing enterprise density, and enterprise size density on the male and female cancer probabilities.

Since the dependent variable (cancer probability) is a probability-type variable, we use a binomial distribution with a logit link function and apply an independent correlation structure to address the correlation of panel data. Year-fixed effects are controlled for to eliminate potential time trend effects, and robust standard errors are used for estimation.

From the regression results with the changed estimation model, it is evident that, regardless of whether control variables are included, pollution enterprise density, manufacturing enterprise density, and enterprise size density all have a significant positive effect on the cancer probability for both males and females. Under the control variable condition, by converting log-odds (logarithmic odds) and odds ratios (OR), it is found that: pollution enterprise density, manufacturing enterprise density, and enterprise size density all significantly increase male and female cancer incidence rates. The regression results show that for every increase of 1 enterprise per km^2^ in pollution enterprise density, the odds ratio for male cancer incidence increases by 9.8%, and for female cancer incidence, the odds ratio increases by 9.0%. For every increase of 1 enterprise per km^2^ in manufacturing enterprise density, the risk of male cancer incidence increases by 10.8%, and the risk for females increases by 10.0%. For every increase of 1 enterprise per km^2^ in enterprise size density, the risk of male cancer incidence increases by 5.6%, and the risk for females increases by 5.4%. All these estimated results are statistically significant at the 1% level.

#### 4.2.5 Adding control variables

To ensure the robustness of our findings, we account for three key factors that may influence regional cancer incidence: population density, economic activity intensity, and extreme weather conditions. Previous studies have shown that extreme temperatures can modify the air pollution-health relationship (25). For our robustness checks, we include: (1) population density (persons per square kilometer), (2) DMSP-OLS nighttime light imagery as a proxy for economic activity intensity (3) meteorological controls using the 90th percentile of daily temperature (extreme heat) and 95th percentile of rainfall (extreme precipitation) (26). The weather indicators were calculated from daily station observations by counting provincial-level days exceeding these thresholds. The results demonstrate consistent robustness after incorporating these additional controls.

The above robustness regression results are shown in Table 4.

**Table 4:** Estimation results of robustness checks (2).

**Changing the model**	**(1)**	**(2)**	**(3)**	**(4)**
	**MCP**	**MCP**	**FCP**	**FCP**
PED	0.080^***^	0.094^***^	0.081^***^	0.086^***^
	(4.755)	(5.723)	(4.155)	(4.320)
MED	0.092^***^	0.103^***^	0.093^***^	0.095^***^
	(5.412)	(6.215)	(4.667)	(4.685)
MED	0.042^***^	0.054^***^	0.048^***^	0.053^***^
	(4.622)	(5.922)	(4.568)	(4.861)
Time fixed effects	Y	Y	Y	Y
Region fixed effects	Y	Y	Y	Y
Control variables included	N	Y	N	Y
*N*	1,797	1,797	1,797	1,797
(Adding control variables)		MCIR		FCIR
PED		11.295^**^		6.557^*^
		(2.266)		(1.869)
MED		13.114^**^		7.563^**^
		(2.414)		(2.011)
ESD		6.032^**^		3.596^*^
		(2.093)		(1.650)
Time fixed effects		Y		Y
Region fixed effects		Y		Y
Control variables included		Y		Y
*N*		1,797		1,797

^***^p < 0.01, ^**^p < 0.05, ^*^p < 0.1.

### 4.3 Heterogeneity analysis

#### 4.3.1 Detailed cancer types

This study selects the five most prevalent types of cancer in China from 2008 to 2016—tracheobronchial lung cancer, stomach cancer, liver cancer, colorectal cancer, and female breast cancer—to further explore the impact of polluting enterprises on regional cancer incidence rates. The regression results are shown in Table 5. Columns (1), (3), (5), and (7) display the regression results for the effect of pollution enterprise density on male tracheobronchial lung cancer, stomach cancer, liver cancer, and colorectal cancer incidence rates, respectively. Columns (2), (4), (6), (8), and (9) show the regression results for the effect of pollution enterprise density on female tracheobronchial lung cancer, stomach cancer, liver cancer, colorectal cancer, and breast cancer incidence rates.

**Table 5:** Estimation results for detailed cancer types.

**Variable**	**(1)**	**(2)**	**(3)**	**(4)**	**(5)**	**(6)**	**(7)**	**(8)**	**(9)**
	**Tracheobronchial lung cancer**		**Stomach cancer**		**Liver cancer**		**Colorectal cancer**		**Breast cancer**
PED	2.579^**^	2.536^**^	1.221	1.209^*^	1.886^**^	−0.440	0.338	0.173	0.562
	(2.007)	(2.318)	(0.882)	(1.710)	(2.101)	(−1.099)	(0.321)	(0.224)	(0.625)
Time fixed effects	Y	Y	Y	Y	Y	Y	Y	Y	Y
Region fixed effects	Y	Y	Y	Y	Y	Y	Y	Y	Y
Control variables included	Y	Y	Y	Y	Y	Y	Y	Y	Y
*N*	1,797	1,797	1,797	1,797	1,797	1,797	1,797	1,768	1,797

^***^p < 0.01, ^**^p < 0.05, ^*^p < 0.1.

As shown in Table 5, the regression results indicate that pollution enterprise density has a significant positive impact on both male and female tracheobronchial lung cancer incidence rates at the 5% significance level. Specifically, for every increase of 1 enterprise per km^2^ in regional pollution enterprise density, the male and female tracheobronchial lung cancer incidence rates increase by 2.579 (per 100,000 people) and 2.536 (per 100,000 people), respectively. Additionally, pollution enterprise density has a significant positive effect on male liver cancer incidence at the 5% significance level. For every increase of 1 enterprise per km^2^ in pollution enterprise density, the male liver cancer incidence rate increases by 1.886 (per 100,000 people). For female stomach cancer, pollution enterprise density shows a positive effect at the 10% significance level. For every increase of 1 enterprise per km^2^ in pollution enterprise density, the female stomach cancer incidence rate increases by 1.209 (per 100,000 people). This regression result also provides an explanation for the fact that lung cancer has consistently ranked as the leading cancer incidence in China in recent years.

The regression results of this section are shown in Table 5.

#### 4.3.2 Division into four major regions

Considering the regional differences in the distribution of polluting enterprises, this study divides the samples into the Eastern, Central, Western, and Northeastern regions for analysis. First, a heatmap analysis is conducted, which reveals that when the pollution enterprise density is between intervals 3 and 4, both male and female cancer incidence rates are generally higher, indicating a positive correlation between pollution enterprise density and cancer incidence rates. Additionally, the cancer incidence rates show a trend where the Eastern and Northeastern regions have higher rates, while the Central and Western regions have lower rates.

**Figure 2:**
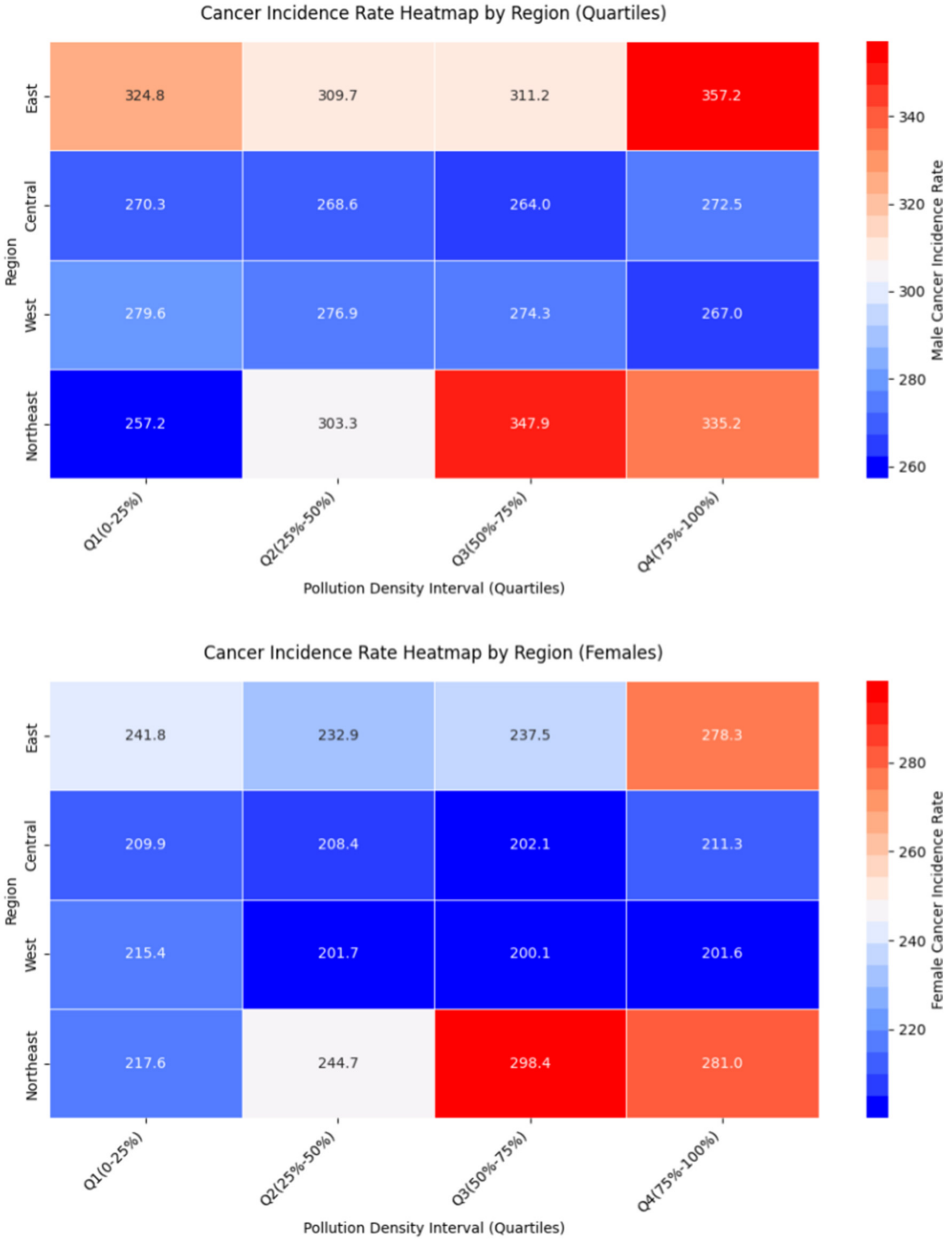
Heatmap analysis of the four main sectors.

The above heat map analysis is shown in Figure 2.

Next, the regression of Equation 1 is conducted separately by region, and the results are shown in Table 6. The odd-numbered columns display the regression results for male overall cancer incidence rates, and the even-numbered columns show the results for female cancer incidence rates. Table 6 indicates that pollution enterprise density, manufacturing enterprise density, and pollution enterprise size density have a significant positive effect on the cancer incidence rates for males and females in the Eastern region, and on the male cancer incidence rate in the Northeastern region, all at the 1% significance level. For female cancer incidence in the Northeastern region, the effect is positive at the 10% significance level. In the Central and Western regions, the effects of pollution enterprise density and manufacturing enterprise density on male cancer incidence are not significant, and for female cancer incidence in the Central region, the effect is also not significant. Furthermore, it is found that in the Western region, manufacturing enterprise density and enterprise size density have a significant negative impact on the overall cancer incidence rate for females.

**Table 6:** Analysis of regional heterogeneity.

**Variable**	**(1)**	**(2)**	**(3)**	**(4)**	**(5)**	**(6)**	**(7)**	**(8)**
	**Eastern**		**Central**		**Western**		**Northeastern**	
PED	23.498^***^	18.248^***^	−0.029	0.388	−5.468	−10.512	42.348^***^	24.183^*^
	(2.934)	(3.237)	(−0.006)	(0.075)	(−0.484)	(−1.395)	(2.923)	(1.893)
MED	23.829^***^	18.968^***^	−0.897	−0.727	−6.849	−14.035^*^	46.758^*^	24.478^*^
	(2.936)	(3.313)	(−0.138)	(−0.128)	(−0.569)	(−1.698)	(3.585)	(1.795)
ESD	17.066^***^	13.763^***^	−2.411	−0.695	−6.357	−7.788^*^	25.107^***^	14.233^*^
	(4.297)	(3.984)	(−0.653)	(−0.234)	(−0.943)	(−1.788)	(3.143)	(1.114)
Time fixed effects	Y	Y	Y	Y	Y	Y	Y	Y
Region fixed effects	Y	Y	Y	Y	Y	Y	Y	Y
Control variables included	Y	Y	Y	Y	Y	Y	Y	Y
*N*	695	695	508	508	425	425	169	169

^***^p < 0.01, ^**^p < 0.05, ^*^p < 0.1.

The regression results of this section are shown in Table 6.

Regional differences explanation:

The Eastern region, as the most economically developed area in China, has a dense distribution of polluting enterprises, which are generally larger in scale and form regional clusters of polluting industries. This high density of polluting enterprises intensifies the environmental pollution burden, posing a significant threat to residents' health.

Due to historical development factors, the Northeastern region has the second-highest pollution enterprise density. During the planned economy period, the three Northeastern provinces established heavy industries such as steel, chemicals, machinery, and papermaking. Although the economic growth in the Northeast slowed between 2000 and 2008, industrial restructuring has not been completed, and traditional heavy industries remain dominant, contributing to higher pollution enterprise density. Similar to the Eastern region, the environmental issues caused by these polluting enterprises have had a significant impact on public health, leading to higher cancer incidence rates nationwide.

In the Central region, polluting enterprises are primarily concentrated in resource-based cities and industrial bases. Although the number of enterprises is large, their distribution is more scattered. The Central region undertook part of the coastal industrial transfer during the 2000s, leading to rapid development in industries like steel, chemicals, and machinery manufacturing, but industrial agglomeration remains relatively low. The urbanization and population density are also lower than in the Eastern region, so the health impact of pollution enterprise density is relatively limited.

In the Western region, due to lower economic development levels and weaker industrial foundations, pollution enterprise density is significantly lower than in the Eastern and Central regions. The Chengdu-Chongqing area, although having some industrial infrastructure, has polluting enterprises concentrated mainly in a few industrial parks, limiting the overall impact. The Northwest region, dominated by resource-based industries such as coal, petrochemicals, and metallurgy, has a low population density and lower urbanization levels, resulting in a relatively smaller health burden from pollution. For the Western region, the explanation for the significant negative impact of manufacturing enterprise density and enterprise size on female cancer incidence is that manufacturing enterprises, compared to the overall polluting enterprises (which include resource-consuming industries like mining), have more long-term economic effects. Additionally, enterprise size, which represents the economic effects of enterprises, is likely to foster economic development and infrastructure improvements, as well as enhance medical levels. These measures improve public health, thereby reducing cancer incidence risks in the Western region.

**Figure 3:**
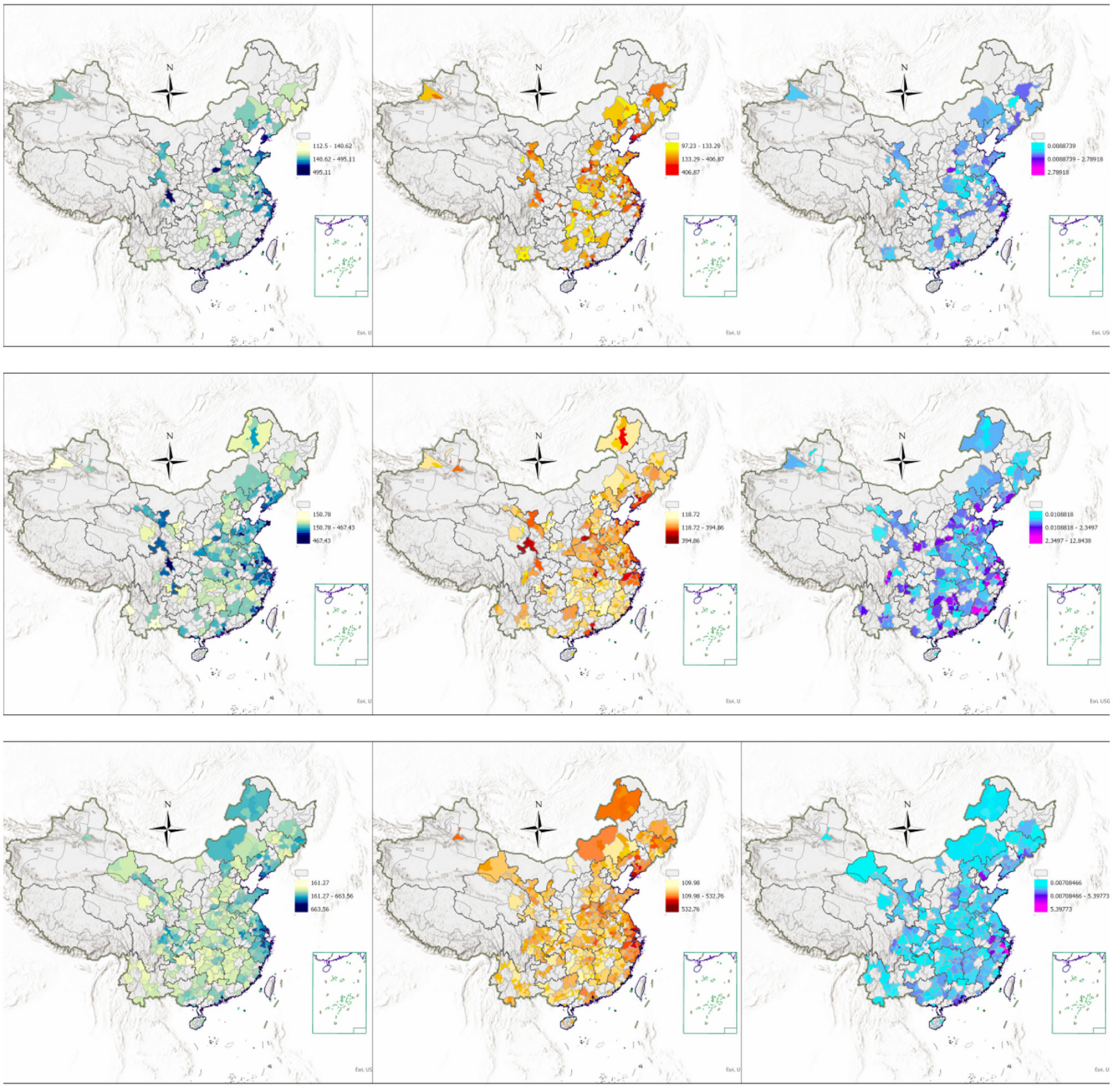
Cancer incidence rates at registration sites and pollution density map.

The incidence rates of male (first column) and female (second column) at the registration points in 2002, 2005 and 2008, and the density of polluting enterprises in 2010, 2013 and 2016 (third column) are shown in Figure 3.

The images were created by the author using ArcGIS Pro.

Gender differences explanation:

Although there is research have shown that both men and women are susceptible to the risks of air pollution (27), This study shows that industrial pollution exerts a disproportionately higher cancer burden on male populations, a disparity attributable to two synergistic mechanisms: first, gender-based occupational segregation leads to males predominantly engaging in high-exposure outdoor occupations (e.g., manufacturing, construction), while females are disproportionately concentrated in lower-exposure indoor service sectors (28). Second, the employment structure of polluting enterprises itself exhibits significant gender disparity, with male workers constituting 72–85% of the workforce in heavy industries according to ILO statistics (2022). This dual exposure pathway—through both residential environmental.

#### 4.3.3 Coastal and inland division

Given that industrialization and urbanization in China have followed a pattern of coastal development first, followed by inland development, this study divides the samples based on whether the registry point's province is located on the coast. The regression of Equation 1 is then conducted separately by region, and the results are shown in Table 7. Odd-numbered columns represent the regression results for male overall cancer incidence rates, while even-numbered columns represent the results for females. The results in Table 7 show that in coastal regions, pollution enterprise density, manufacturing enterprise density, and pollution enterprise size density have a significant positive impact on male and female overall cancer incidence rates at the 1% significance level. In inland regions, pollution enterprise density, manufacturing enterprise density, and enterprise size density do not significantly affect male or female overall cancer incidence rates. It can be observed that the impact of polluting enterprises on cancer incidence is more significant in China's coastal regions compared to inland regions.

**Table 7:** Analysis results of coastal vs. inland division.

**Variable**	**(1)**	**(2)**	**(3)**	**(4)**
	**Coastal region**		**Inland region**	
PED	18.551^***^	12.293^**^	6.436	1.931
	(2.740)	(2.493)	(0.838)	(0.337)
MED	19.032^***^	13.171^***^	8.370	1.112
	(2.755)	(2.626)	(0.876)	(0.163)
ESD	13.102^***^	9.208^***^	0.746	−0.771
	(3.527)	(2.853)	(0.177)	(−0.249)
Time fixed effects	Y	Y	Y	Y
Region fixed effects	Y	Y	Y	Y
Control variables included	Y	Y	Y	Y
*N*	838	838	959	959

^***^p < 0.01, ^**^p < 0.05.

This is because, between 2000 and 2008, most of the polluting enterprises in China were concentrated in coastal regions. For instance, the rapid development of the manufacturing industry in the southern Jiangsu region of the Yangtze River Delta led to a dense concentration of chemical, dyeing, and electronic enterprises. The Pearl River Delta, including Dongguan, Shenzhen, and Foshan, became global manufacturing hubs with widespread distribution of textile, electronics, metal processing, and chemical enterprises. In the Bohai Rim region, chemical, papermaking, and pharmaceutical industry clusters were formed along the coastline in areas such as Weifang, Zibo, and Binzhou in Shandong. Additionally, Tangshan in Hebei and the Tianjin Binhai New Area had concentrated steel and chemical industries. Therefore, during this period, many polluting enterprises were concentrated along the coastal areas, leading to higher levels of environmental pollution and greater health risks for coastal residents.

As environmental regulations in coastal regions gradually strengthened, some high-polluting enterprises began shifting to the Central and Western inland regions after 2008, where environmental governance costs were lower and environmental carrying capacity was relatively stronger. For example, industries gradually moved to Jiangxi, Anhui, and Henan, but during the 2000–2008 period, inland regions still had relatively fewer polluting enterprises.

The regression results of this section are shown in Table 7.

#### 4.3.4 Breakpoint division

To investigate the potential threshold effect of polluting enterprises on cancer incidence, this study employs the Bootstrap method with 2,000 resampling iterations. The Bootstrap approach was selected for its ability to robustly quantify uncertainty in threshold estimation, providing reliable confidence intervals for these critical breakpoints. Our analysis reveals distinct thresholds: 1.0811 (95% CI: [0.9224, 1.1399]) for males and 0.4286 (95% CI: [0.4002, 0.5070]) for females. These breakpoints, which identify significant shifts in cancer risk across pollution levels, are subsequently used to conduct grouped regression analyses. Table 8 presents these findings, with odd-numbered columns showing male cancer incidence results and even-numbered columns displaying female outcomes.

**Table 8:** Breakpoint division analysis results.

**Variable**	**(1)**	**(2)**	**(3)**	**(4)**
	**Before breakpoint**		**After breakpoint**	
PED	−1.053	−21.408	23.733^**^	13.848^***^
	(−0.123)	(−0.967)	(2.156)	(3.364)
MED	0.452	−17.433	26.918^**^	14.851^***^
	(0.046)	(−0.802)	(2.345)	(3.380)
ESD	−4.190	−21.871^**^	14.851^**^	7.707^***^
	(−0.707)	(−2.159)	(2.252)	(3.027)
Time Fixed Effects	Y	Y	Y	Y
Region Fixed Effects	Y	Y	Y	Y
Control Variables Included	Y	Y	Y	Y
*N*	1,439	810	358	987

^***^p < 0.01, ^**^p < 0.05.

**Figure 4:**
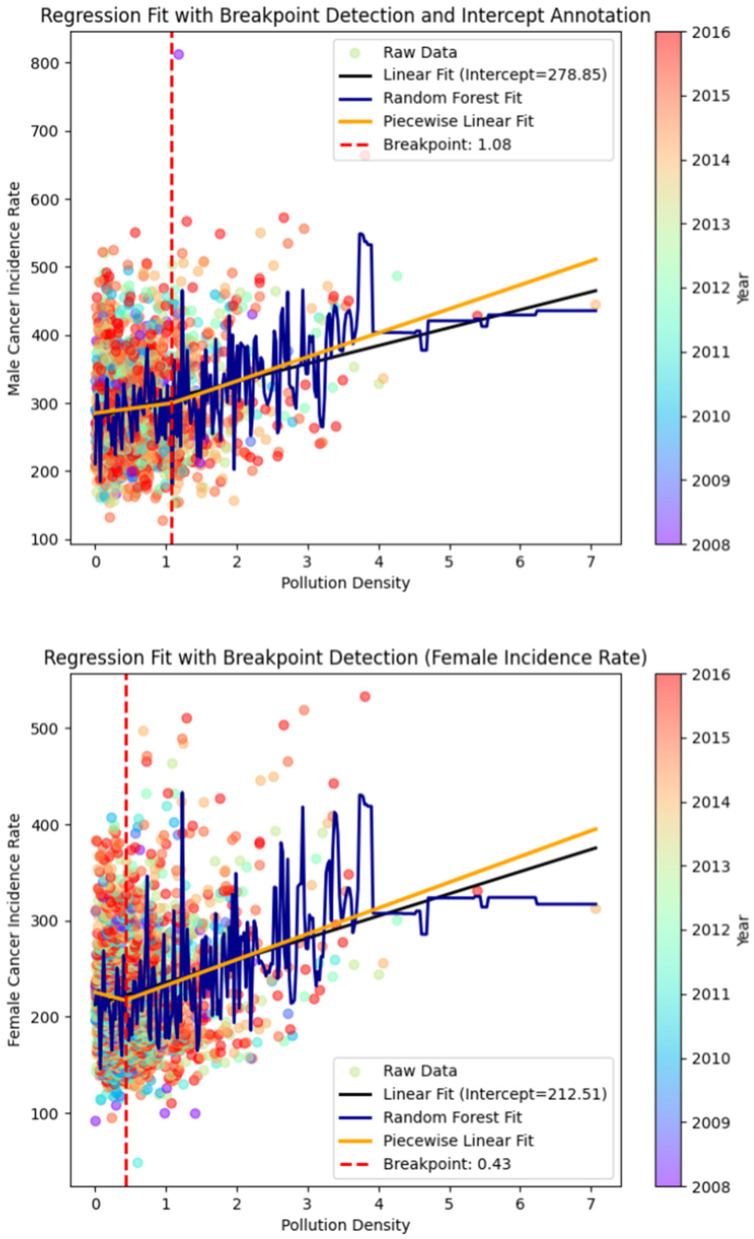
Machine learning breakpoint diagram.

The machine learning breakpoint graphs of pollution density on the incidence rates of men and women are shown in Figure 4.

The results show that, prior to the pollution density breakpoint, the effects of pollution enterprise density and manufacturing enterprise density on overall cancer incidence rates are not significant, and the effect of pollution enterprise size density on female cancer incidence is significantly negative. However, after the pollution density breakpoint, pollution enterprise density, manufacturing enterprise density, and enterprise size density all exhibit significant positive effects on overall cancer incidence rates, and all passed the 1% or 5% significance tests.

The regression results suggest that when regional pollution enterprise density is within a relatively small range, the effect of polluting enterprises on cancer incidence is not significant. Moreover, the economic benefits brought by polluting enterprises could potentially reduce the regional cancer incidence rate. However, when pollution enterprise density reaches a certain level, the impact of polluting enterprises on cancer incidence becomes particularly significant. Therefore, it is crucial to control the regional pollution enterprise density at a reasonable level through policy regulations.

The regression results of this section are shown in Table 8.

#### 4.3.5 Environmental regulation intensity division

To demonstrate the importance of environmental regulations in influencing the impact of polluting enterprises on residents' health, this section conducts a heterogeneity analysis based on environmental regulation intensity. Python software is used for large-scale text analysis of government work reports to obtain the frequency of keywords related to environmental regulations (such as environmental protection, pollution, energy consumption, emission reduction, wastewater discharge, ecology, green, low carbon, air quality, chemical oxygen demand, sulfur dioxide, carbon dioxide, PM10, and PM2.5) in the majority of local government reports across the country (29). Based on the total word frequency, regions are categorized into high and low environmental regulation areas.

From the regression results in Table 9, it is evident that the intensity of environmental regulations significantly affects the impact of polluting enterprises on cancer incidence rates. In regions with high environmental regulation, pollution enterprise density, manufacturing enterprise density, and enterprise size density do not reach statistical significance. This indicates that, under stronger environmental regulations, the impact of polluting enterprises on cancer incidence rates is effectively suppressed. In contrast, in regions with low environmental regulation, the regression coefficients for pollution enterprise density, manufacturing enterprise density, and enterprise size density are all significantly positive at the 1% or 5% significance level. This suggests that, in a more relaxed regulatory environment, the presence of polluting enterprises significantly increases cancer incidence rates, with a clear impact on both males and females. This demonstrates that, when environmental regulation is weak, pollution emissions are not effectively controlled, exacerbating the health damage to residents.

**Table 9:** Analysis results For regulation intensity classification.

**Variable**	**High regulation areas**		**Low regulation areas**	
PED	8.974	8.390	26.508^***^	16.075^***^
	(1.199)	(1.283)	(2.964)	(2.951)
MED	11.486	10.173	28.708^***^	16.558^***^
	(1.195)	(1.282)	(3.232)	(3.213)
ESD	6.236	6.145	12.570^**^	7.514^**^
	(1.534)	(1.584)	(2.403)	(2.203)
Time fixed effects	Y	Y	Y	Y
Region fixed effects	Y	Y	Y	Y
Control variables included	N	Y	N	Y
*N*	817	817	849	849

^***^p < 0.01, ^**^p < 0.05.

Strict environmental policies and governance measures can reduce the negative health impacts of industrial pollution and slow the cancer incidence risks caused by polluting enterprises. Clearly, China's government environmental regulations are effective: strict environmental protection policies, pollution control measures, and corporate compliance requirements likely reduce the diffusion of pollutants and decrease health risks for residents. However, in areas with weaker environmental regulations, pollution problems remain severe, posing a significant threat to public health.

The regression results of this section are shown in Table 9.

### 4.4 Endogeneity analysis

When analyzing the impact of polluting enterprises on potential cancer incidence, the endogeneity issue does not appear to be significant. However, when further exploring the impact of polluting enterprises on environmental pollution, endogeneity becomes an unavoidable concern. Specifically, on one hand, polluting enterprises may exacerbate environmental degradation through the emission of harmful substances; on the other hand, polluting enterprises may also have an incentive to establish factories in areas with severe pollution due to factors such as environmental policy regulations and pollution control costs.

This study uses the historical industrial layout of regions, specifically the pollution enterprise density in 1992, as an instrumental variable. Pollution enterprise data from Qichacha was used to identify the pollution enterprises that were established before or in 1992 and were operational in 1992, allowing for the calculation of the pollution enterprise density in each region.

The choice of 1992 as the key time point is based on the fact that the “Southern Talk” of 1992 marked a significant deepening of China's market reforms, leading to a substantial acceleration of the national industrialization process. Before 1992, polluting enterprises were mainly concentrated within the state-owned industrial system, and site selection was policy-driven. After 1992, the market economy reforms accelerated, with more private and foreign-invested enterprises entering the market. As a result, the distribution of polluting enterprises in various regions was influenced by the industrial foundation before 1992. Therefore, the use of the 1992 pollution enterprise distribution as an instrumental variable ensures both the relevance and exogeneity of the data.

To ensure that the instrumental variable is appropriate, two core conditions must be met: relevance and exogeneity. The pre-1992 pollution enterprise layout exhibits significant path dependence (30) and industrial agglomeration effects (31). The distribution of polluting enterprises before 1992 largely determined the location of subsequent pollution enterprises. Existing polluting enterprises attracted investment in upstream and downstream industries, drawing additional polluting enterprises to the same region. Moreover, these enterprises often relied on existing infrastructure, supply chains, and policy environments, resulting in industrial continuity (32). Regarding exogeneity, the pollution enterprises before 1992 did not directly affect current PM2.5 concentrations: PM2.5 is primarily determined by recent pollution emissions, while most of the pollution enterprises before 1992 have either closed, undergone technological upgrades, or modified their emission patterns due to government environmental policies and technological advancements. As a result, the historical emissions of these enterprises do not directly influence current PM2.5 levels. Therefore, the pollution enterprise distribution of 1992 only indirectly influences current pollution enterprise layout, which in turn affects PM2.5, but does not directly influence PM2.5 levels. This means the pollution enterprise distribution of 1992 meets the exogeneity requirement for an instrumental variable.

In summary, to address the potential reverse causality between polluting enterprises and environmental pollution, this study uses the instrumental variable approach and constructs a two-stage least squares (2SLS) regression model as follows:


(4) $$\begin{array}{l} \text{PollutingFirm}{\text{s}}_{\text{it}}=\text{α}+{\text{β}}_{1}\text{PastPollutingFirm}{\text{s}}_{\text{i},\text{pre}-1992}\\ \;+{\text{β}}_{2}\text{Control}{\text{s}}_{\text{it}}+{\text{μ}}_{\text{i}}+{\text{v}}_{\text{t}}+{\text{ϵ}}_{\text{i},\text{t}} \end{array}$$

(5) $$\begin{array}{l} \text{PM}2.5_{\text{it}}=\text{γ}+{\text{δ}}_{1}{\hat{\text{PollutingFirms}}}_{\text{it}}+{\text{δ}}_{2}\text{Control}{\text{s}}_{\text{it}}\\ \;+{\text{μ}}_{\text{i}}+{\text{v}}_{\text{t}}+{\text{ϵ}}_{\text{i},\text{t}} \end{array}$$

As shown in the regression results, in the first-stage regression, we use historical industrial density as an instrumental variable for pollution enterprise density. The regression results indicate that this variable has a significant effect on pollution enterprise density, with an F-statistic of 12.22 in the first stage, which is well above the empirical threshold of 10. This effectively rules out the “weak instrument” problem. This suggests that our instrumental variable has strong explanatory power and can effectively identify the exogenous variation in pollution enterprise density.

In the second-stage regression, we find that pollution enterprise density has a significant effect on the average PM2.5 concentration. The overall F-statistic for the regression model is 44.93, indicating that the regression model is statistically significant. This shows that polluting enterprises have a significant impact on regional environmental pollution, and the direction of the effect is in line with expectations.

Specifically, the first-stage regression results show that for every increase of 1 enterprise per km^2^ in historical industrial density, the pollution enterprise density increases by 0.391 enterprises per km^2^. Correspondingly, the second-stage regression results show that for every increase of 1 enterprise per km^2^ in pollution enterprise density, the annual average PM2.5 concentration in the region increases by 1.889 micrograms per cubic meter.

Regarding control variables, the first-stage regression shows that the average area of crop cultivation has a significantly negative impact on the location of polluting enterprises, while the average area of water bodies and undeveloped urban land have a significantly positive impact on the location of polluting enterprises, indicating factors considered in the site selection of polluting enterprises. In the second-stage regression, the average area of crop cultivation, green space, forests, and water body coverage all have a significantly negative effect on reducing the region's PM2.5 concentration. This indicates that lands with more vegetation coverage indeed reduce the concentration of inhalable pollutants. On the other hand, undeveloped land, which is often barren and lacks vegetation, has a significantly positive effect on PM2.5 levels.

The regression results of this section are shown in Table 10.

**Table 10:** Instrumental variable estimation.

**Variable**	**First-stage (Pollution enterprise density)**	**Second-stage (Average PM2.5)**
Historical industrial layout (IV)	0.391^***^ (0.062)	
PED		1.889^**^ (0.913)
perCropland	−0.001^*^ (0.001)	−0.096^***^ (0.011)
perForest	0.000 (0.000)	−0.036^***^ (0.004)
perGrassland	−0.000 (0.000)	−0.024^***^ (0.005)
perWater	0.007^**^ (0.003)	−0.247^***^ (0.087)
perImpervious	0.017^**^ (0.007)	1.401^***^ (0.110)
F-Statistic	12.22	44.93
Constant term	0.201^**^ (0.083)	42.554^***^ (1.509)
Time fixed effects	Y	Y
Region fixed effects	Y	Y
*N*	1,797	1,796
R-squared	0.366	0.295

^***^p < 0.01, ^**^p < 0.05, ^*^p < 0.1, (Robust standard errors).

### 4.5 Mechanism analysis

Since people's daily activities mainly occur near the surface, from the perspective of epidemiology and environmental health research, surface PM2.5 concentrations more accurately reflect the true exposure to pollution levels. Compared to atmospheric PM2.5 concentrations, surface PM2.5 concentrations have a more significant impact on human health, particularly in the incidence of chronic diseases such as cancer.

First, a correlation analysis is conducted between pollution enterprise density, cancer incidence rates, and surface PM2.5 concentrations for the sample registry points (with pollution enterprise density logged and surface PM2.5 concentrations normalized). The results suggest a potential positive correlation between the three variables.

Further, a two-way fixed panel regression model is used for empirical analysis. The regression results compare the effects of non-polluting enterprises and polluting enterprises on cancer incidence rates. The density of non-polluting enterprises does not have a significant impact on surface PM2.5 concentrations, regardless of whether regional environmental control variables are included. This result suggests that the presence of non-polluting enterprises does not have a substantive effect on changes in surface PM2.5 concentrations. In contrast, the density of polluting enterprises shows a significant positive effect on the annual average surface PM2.5 concentrations, and at the 10% significance level, pollution enterprises significantly contribute to the rise in surface PM2.5 concentrations. The greater the density of polluting enterprises, the higher the PM2.5 concentration in the region, which may further exacerbate the air pollution problem.

To further investigate the impact of polluting enterprises on environmental pollution, this study classifies polluting enterprises into heavy polluting enterprises and light polluting enterprises based on industry classification codes. Based on the regression results provided in the table, the impact of the density of heavy polluting and light polluting enterprises on surface PM2.5 is further analyzed.

From the regression results, density of heavy polluting enterprises has a significant positive effect on surface PM2.5, and this effect is significant at the 5% level. In contrast, the density of light polluting enterprises does not have a significant effect on surface PM2.5 concentrations. This indicates that light polluting enterprises contribute less to changes in surface PM2.5 concentrations and do not have a noticeable impact on air pollution.

Overall, the regression results suggest that the density of heavy polluting enterprises has a significant effect on surface PM2.5 concentrations, while light polluting enterprises do not show a significant impact. This analysis further reveals the different effects of enterprises with varying levels of pollution on regional air quality, providing valuable insights for policymakers in addressing pollution from enterprises.

A growing body of meta-analyses have found that long-term exposure to PM2.5 will markedly increase the incidence of lung cancer, breast cancer, liver cancer, colorectal cancer and other cancers (8, 9, 33, 34).

The above regression results are shown in Table 11.

**Table 11:** Mechanism analysis results.

	**(1)**	**(2)**
	**Surface PM2.5**	
NPED	0.000	−0.000
	(0.082)	(−0.091)
PED	0.095^*^	0.097^*^
	(1.694)	(1.767)
HPED	0.173^**^	0.180^**^
	(2.089)	(2.209)
LPED	0.007	0.002
	(0.089)	(0.028)
Time fixed effects	Y	Y
Region fixed effects	Y	Y
Control variables included	N	Y
*N*	1,762	1,762

^***^p < 0.01, ^**^p < 0.05, ^*^p < 0.1.

### 4.6 Medical compensation differences

This section further analyzes the heterogeneous impact of polluting enterprises on healthcare levels in urban and county-level areas. The regression results show that the density of non-polluting enterprises does not significantly affect healthcare levels in either urban or county areas. This suggests that the presence of non-polluting enterprises does not have a significant impact on healthcare levels in both types of regions. However, polluting enterprises have a significant positive effect on healthcare levels in urban areas, indicating that these polluting enterprises contribute to improving healthcare levels in urban regions. In contrast, in county-level areas, the presence of polluting enterprises does not significantly affect healthcare levels. Polluting enterprises significantly improve healthcare levels in urban areas but fail to have the same effect in county areas.

In summary, the regression results indicate that the impact of polluting enterprises on healthcare levels varies by region. Specifically, in urban areas, polluting enterprises can significantly improve healthcare levels, whereas in county areas, they fail to achieve the same result. This disparity can be explained by the differences between urban and rural areas in China (35). Local governments, based on local economic conditions and resource disparities, may provide compensation for the health risks posed by polluting enterprises. The differences in medical compensation between urban and rural areas could play an important role in governmental decision-making. The increased fiscal revenue from polluting enterprises may be used to improve healthcare in urban areas, effectively achieving medical compensation for the pollution. In county areas, however, there is less noticeable medical compensation from polluting enterprises, leading to differences in healthcare compensation between urban and rural areas. This regression result provides valuable insights into the unequal distribution of healthcare resources across regions.

The regression results of this section are shown in Table 12.

**Table 12:** Medical compensation mechanism analysis results.

	**(1)**	**(2)**
	**Urban areas**	**County areas**
NPED	−0.243	0.488
	(−0.355)	(1.451)
PED	2,635.666^**^	−29.233
	(2.187)	(−0.597)
MED	2,834.839^**^	−12.528
	(2.213)	(−0.234)
HPED	2,659.583^*^	−39.006
	(1.719)	(−0.620)
LPED	6,548.361^**^	−30.718
	(1.999)	(−0.288)
Time fixed effects	Y	Y
Region fixed effects	Y	Y
Control variables included	Y	Y
*N*	1,058	724

^**^p < 0.05, ^*^p < 0.1.

## 5 Discussion

### 5.1 Key findings

Research into the impact of polluting enterprises on cancer incidence is crucial. This study focuses on the environmental “legacy” of China's rapid industrialization period from 2000 to 2008, exploring the relationship between the presence of polluting enterprises and cancer incidence rates. By constructing econometric models and utilizing data from Qichacha and the *China Cancer Registry Annual Report*, this study provides an in-depth analysis of the impact of polluting enterprises on cancer incidence and its specific mechanisms. This study finds the following:

1. Polluting enterprises significantly increase the cancer incidence rates for both males and females, especially for lung cancer, exacerbating the public health burden.
2. This impact is more pronounced in the Eastern region, followed by the Central and Northeastern regions, with the Western region showing minimal effects. Additionally, the impact is more significant in coastal areas than in the inland areas.
3. There may be a threshold effect in the relationship between pollution enterprise density and cancer incidence: when pollution enterprise density is below a certain threshold, it may not have an impact on cancer incidence and may even reduce the impact on cancer incidence due to local economic development. However, when pollution enterprise density exceeds a certain threshold, it positively affects cancer incidence. Therefore, strict environmental policies and regulations are especially important.
4. Local government environmental regulations can reduce the negative impact of industrial pollution on public health, mitigating the cancer incidence risks associated with polluting enterprises.
5. The impact of polluting enterprises on male cancer incidence is greater than on female cancer incidence, which may be related to gender differences in occupational choices.
6. Polluting enterprises significantly increase local environmental burdens, by raising PM2.5 concentrations and consequently affecting the health of regional residents.
7. There are medical compensation differences between urban and rural areas: polluting enterprises can significantly improve healthcare levels in urban areas but have no significant impact on county-level areas.

### 5.2 Policy recommendations

It is worth noting that China's environmental and public health policies in the post-rapid-development stage offer valuable lessons for other developing countries. In 2008, the Chinese government elevated environmental protection to a national strategic priority by upgrading the State Environmental Protection Administration to the ministerial-level Ministry of Environmental Protection, and by issuing the White Paper on China's Policies and Actions for Addressing Climate Change, which integrated energy conservation, greenhouse gas emissions control, and renewable energy development into the national policy framework. In recent years, China has further intensified its efforts in public health and pollution governance. For example, in 2016, the government launched the Healthy China 2030 Planning Outline, which aims to increase the average life expectancy to 79 years by 2030, with a strong emphasis on chronic disease prevention and the promotion of healthier lifestyles. To achieve this goal, the government has implemented a range of measures, including strengthening health education, advocating healthy lifestyles, and improving the healthcare service system.

In terms of pollution control, the Chinese government has strengthened the governance of polluting enterprises by establishing the “Three Lines and One List” ecological environment zoning control system, dividing national land space into priority protection, key control, and general control units, and implementing differentiated access mechanisms. In terms of legal reform, the revision of the Environmental Protection Law established the principle of lifelong accountability for polluters. The Soil Pollution Prevention and Control Law of 2020 clarified the corporate responsibility, forming a full-chain responsibility system of “pollution accountability—risk prevention—ecological restoration.” On the economic front, the implementation of dynamic environmental taxes and green credit policies has translated the health benefits of enterprises that have undergone ultra-low emission transformations into tax reductions, using market mechanisms to push enterprises toward technological upgrades. Data from 2024 show that the national average PM2.5 concentration has decreased by 36% compared to 2015, with pollution reduction achieved alongside a 63% growth in GDP, confirming the effectiveness of the “development and protection collaboration” governance path.

Based on the empirical findings and recent policy practices in China, the following policy recommendations are proposed for China as well as other emerging economies and rapidly developing countries, with the aim of advancing coordinated environmental and public health governance in the context of industrialization.

First, public health outcomes should be integrated into environmental oversight frameworks to establish a comprehensive system linking environmental quality, emission intensity, and population health, thereby enhancing long-term governance responsiveness; second, institutional and financial support for high-pollution enterprise clustering in monitored zones must be strengthened, ensuring advanced pollution treatment infrastructure and stringent environmental standards to mitigate residential spillover effects; third, differentiated governance should be implemented based on local ecological carrying capacities, balancing industrial growth with public health protection through adaptive land-use policies, particularly in environmentally vulnerable regions; finally, routine health monitoring participation must be improved via community-based services, mobile screening, and digital platforms to ensure affordable and convenient preventive care in high-risk areas, ultimately reducing pollution-related health burdens.

### 5.3 Limitations and future prospects

This study has the following limitations:

First, methodological constraints may affect the scientific rigor of this study. Uncontrolled confounding factors in data collection and analysis could compromise the accuracy and reliability of our findings. Second, our multi-level analysis spanning municipal, district, and county regions faced inherent data limitations, including missing values and inconsistent measurement standards across different administrative levels. These constraints were particularly evident in our environmental pollution measures, where we were limited to using PM2.5 concentration as the primary mediator due to data availability issues. Future research would benefit from incorporating additional pollution indicators such as water and soil contamination metrics to provide a more comprehensive assessment. Meanwhile, this article focuses on the impact of polluting enterprises on the overall incidence rate of cancer. The detailed research on specific disease types still needs to be improved. Third, in terms of addressing endogeneity, the onset of cancer involves complex influencing factors, making it difficult to identify variables that fully satisfy the exogeneity condition. This study focuses on the endogeneity issue between environmental pollution and the presence of polluting enterprises. Fourth, the current literature provides insufficient evidence on regional heterogeneity in the health impacts of polluting enterprises. The existing indicator framework lacks the necessary sophistication to adequately capture these spatial variations. Subsequent studies should develop multidimensional metrics to better evaluate the health consequences of industrial pollution across different geographical contexts.

## Data Availability

The original contributions presented in the study are included in the article/supplementary material, further inquiries can be directed to the corresponding author.
